# Monthly Summary

**Published:** 1873-03

**Authors:** 


### MONTHLY SUMMARY.

*Influence of Coffee and Cocoa on Nutrition.*—At a recent meeting of the French Academy of Science, M. Robuteau read an interesting paper on the influence of coffee and cocoa upon nutrition. From the result of experiments which the author made with dogs, he arrived at the conclusion that cocoa and coffee are not mere stimulants like alcohol, but that they directly contribute to the nourishment of the tissues. A dog which was fed daily upon 20 grammes of bread, 10 grammes of fresh butter, and 10 grammes of sugar, died in 29 days, obviously from defective nutrition ; whilst a dog supplied with 20 grammes of cocoa, 10 grammes of sugar, and an infusion of 20 grammes of roasted coffee, was alive and healthy then, though after 29 days.

M. Robuteau stated that the evil results sometime experienced by the continued use of coffee are not felt if the coffee be properly roasted. When the berries are too highly heated, an injurious substance termed caffeine is developed in them.

We believe that a large proportiou of the coffee used in Paris is prepared by subjecting the berries to a current cf heated air or super-heated steam. We wish that this kind of coffee were more frequently to be met with in these countries, for the infusion yielded by these semi-charred berries of the British grocer is often anything but a delectable beverage.

Another French *savant*, M. Charles Gazeau, still more recently communicated to the Academy of Sciences an account of experiments performed upon himself, the results of which appeared to show that cocoa decidedly increased the action of the heart and other vital organs. He believed, however, that the benefits derived from its use are more apparent than real, and that it acted by merely stimulating the vital powers, by causing a more rapid destruction or metamorphosis of tissue. This is the old theory as regards the action of both tea and coffee upon the animal economy. We can, however, hardly doubt the superiority of cocoa over coffee and tea as a merely nutritive principle, for its chemical composition shows that it is rich in fat-forming and muscle-making materials. It is well known, too, that in parts of South America cocoa constitutes a staple article of food amongst the Indian population.—*The Chloralum Review.*

*Mysterious Influences.*—Persons sometimes feel remarkably well—the appetite is vigorous, eating is a joy, digestion vigorous, sleep sound, with an alacrity of body and exhilaration of spirits which altogether throw a charm over life that makes us pleased with everybody and everything. Next week, to-morrow, in an hour, a marvellous change comes over the spirit of the dream , the sunshine has gone, clouds portend, darkness covers the face of the great deep, and the whole man, body and soul, wilts away like a flower without water in midsummer.

When the weather is cool and clear and bracing, the atmosphere is full of electricity ; when it is sultry and moist and without sunshine, it holds but a small amount of electricity, comparatively speaking, and we have to give up what little we have, moisture being a good condnctor; thus, in giving up instead of receiving more, as we would from the cool, pure air, the change is too great, and the whole man languishes. Many becomes uneasy under these circumstances ; "they can't account for it;" they imagine that evil is impending and resort at once to tonics and stimulants, The tonics only increase the appetite, without imparting any additional power to work up the additional food, thus giving the system more work to do, instead of less. Stimulants seem to give more strength ; they wake up the circulation, but it is only temporarily, and unless a new supply is soon taken, the system runs further down than it would have done without the stimulant : hence it is in a worst condition than if none had been taken. The better course would be to rest, take nothing but cooling fruits and berries and melons, and some acid drink when thirsty, adding, if desired, some cold bread and butter ; the very next morning will bring a welcome change.—*Hall's Journal of Health.*

---

*Anchylosis of the Lower Jaw ; Recovery Through the Formation of a False Joint on Both Sides.*—The patient was twenty seven years old, and was affected with complete anchylosis of the jaw, the result of an inflammation following scarlet fever. The growth of both upper and lower jaw was appreciably retarded in consequence of continued disuse. Perfect relief was afforded by an operation, which consisted in removing a wedge-shaped portion of the lower jaw, the base of which pointed downwards. At the time of the report, four months after the operation, the motion of the jaw was completely restored. (Arch. f. Klin. Chirurgie.—*Boston Med. and Surg. Journal.*

---

*Disinfection of Rooms.*—" Among ærial disinfectants chlorine and sulphurous acid are most useful, but neither of these gases can be added to the air of a room in sufficient amounts to destroy the specific poison of small-pox without making the air irrespirable. Organic impurities of all kinds attach themselves to moist surfaces, and sulphurous acid gas seizes with avidity upon everything holding moisture. To completely disinfect a room, including its carpets, furniture, and wall-paper, close the doors, windows, and chimney; put from one to two pounds of brimstone (according to the size of the room) in an iron pot, pour over it a little alcohol, set it on fire, and leave the room for four hours. This process is injurious to the colors of many fabrics, and to gilded articles, and this injury corresponds in degree with the amount of moisture present in the room.

"When disinfection by sulphur fumes is impracticable, the next best thing to do is to wet the carpet, furniture, and walls with a strong solution of carbolic acid, one part in fifty of pure acid.

"Clothing which can be washed may be disinfected by boiling in water for one hour.

"Bedding and clothing which cannot be washed may be disinfected by exposure for four hours to dry heat at a temperature of 225° to 300° Fahr. This can be done in a large brick oven; but every city should have attached to its hospital for infectious diseases an oven specially arranged for the purpose."—*Prof. Derby in Boston Med. & Surg. Journal.*

---

*Anchylosis of the Inferior Maxilla Cured* by the formation of a false joint on each side. (G. Maas: *Arch. f. Klin. Chirurgie, xiii.* B. 3, H.)—A man, 27 years old, had had in his seventh year an attack of scarlatina which left him with an inflammation of the maxillary articulation on both sides, which was followed by complete anchylosis. Both the inferior and superior maxillary bones had suffered in consequence of an arrest of development. Middeldorpf excised on one side, and subsequently Fischer on the other, a wedge-shaped piece from the inferior maxilla, the base of the wedge being downwards, according to Esmarco. The mobility had remained perfect at the time of the report, four months after the operation.—*Med. Times.*

---

*Carbolic Acid as a Local Anæsthetic.*—Carbolic acid possesses wonderful powers as a local anæsthetic. If a portion of skin is covered with a cloth soaked in a saturated solution of carbolic acid for half an hour, and then a streak traced across the surface with a camel's-hair brush dipped in acid liquified by one-twentieth its bulk of water, the skin may be divided along the course of the streak with a sharp scalpel, quite down to the subcutaneous cellular tissue, without causing any pain. Abscesses, whitloes, and buboes may be opened with great advantage in this manner. Sometimes it is necessary, when making an incision through the integument, to brush out the wound made with some liquified acid before making the incision deeper.—*Dr. J. H. Bill, Braithwaite's Retrospect.*

---

*An Ointment for Neuralgia.*—Dr. J. Knox Hodge recommends the following as an application which will relieve facial or any other neuralgia almost instantaneously. He advises his friends to "send around to the druggists and try a bottle"

℞—Albumén of egg, ʒ j.

Oil of peppermint, ℥ iv.

Collodion,

Chloroform, aa ℥ j.—M.

Agitate occasionally for 24 hours, and by gelatinization a beautiful semi-solidified opodeldoc-looking compound results, which will retain its consistency and hold the ingredients intimately blended for months.

In the above we find a local agent of signal potency in all neuralgic affections, and for the relief of pain generally.

Apply by smart friction with the hand, or gently with a soft brush or mop along the course of the nerve involved.—*Georgia Companion.*

---

*The Cure of Stammering—N. Y. Medical Record, Jan. 1st.*—The mode of treatment followed by M. Chervin, of Lyons, in this affection, has lately been the subject of investigation by a commission appointed by the Department Council. The commisioners state that they find the system successful, rapid, and permanent in its effects; which opinion confirsm those of earlier date, given by commissions in France, Belgium, Spain, etc.

Esght patient, severely affected with stuttering, were submitted, under the observation of the commissioners, to the system of M. Chervin. They varied in age from ten to twenty-nine years, and none of them could speak without stammering to an extent most distressing to themselves and to those who heard and saw them In some cases the act of speaking was accompanied with convulsive movements. of the mouth and eyes; in others, with spasmodic respiratory movements. Some had stammered from their infancy; in others the defect had been caused by a shock o the nervous system. Ten days after they had been placedsunder M. Chervin's treatment they were seen by the commissoners, and each of them could then speak distinctly, without stammering er hesitation; and on the 28th they were pronounced cured, speaking then with natural case and rapidity.

The system is as follows. All mechanical contrivances are discarded; but he teaches the patient, by means of a large number of exercises, gradually to pronounce, with distinctness, vowels, consonants syllables, and sentences. He pays great attention to the act of respiration, which he seeks to regulate. He teaches his patients to take at certain intervals a slow but normal inspiration, which is succeeded by an even, continuous, and loud expiration, during which pronunciation is effected. The course of of treatment occupies twenty days, the time being divided into toree periods. During the first the patient is restricted to complete silence, so that the old habit may be broken; duriug the second period the patient is taught ta speak slowly and deliberately; aud during the third period he acquires the practice of speaking fluently and without clipping the words. This method is stated to have succeeded in the most difficult cases, and the good results are said to be permanent; bnt this greatly depends on the patient, who must occasionally make use of the means whico were first used to cure them.—*Med. Times.*

---

*Ingrowing Toe-Nail.*—Dr. Finch writes, that neither of the cutting operations is at all necessary for the complete and rapid cure of ingrowing toe-nail. If a small, thin, flat piece of silver plate bent on one edge into a slight, deep groove, and, after the toe has been poulticed twenty-four hours, slipped beneath the edge of the nail, so as to protect the flesh from its pressure, and the rest of the plate bent round the side and front of the toe, being kept in position with a small portion of resin plaster passed round the toe, a speedy and almost painless cure will take place; and the patient, after the first day, has the additional advantage of being able to walk. I have followed this method in numerous cases with uniform success.—*British Medical Journal.*.

---

*On Longevity.*—Dr. Grey, some years since, published some statistics showing the average age at which the following classes died, after they had attained the age of fifty-one :—

Clergy, 74 years; lawyers, 72¾ yaars; medical men, 73 years; learned professions collectively, 76½ years.

He afterwards added the following observations, which embrace only the most eminent of these professions, with the following results :—

Clergy, (bishops and archbishops) 70¼ years; lawyers, judges, etc., 73 years; medical men, (baronets, etc.) 74½ years.

From his observations he makes the following deductions :— 1. That members of the three learned professions occupy, in respect of the duration of their lives, a favorable position among the educated classes. 2. The difference in the duration of life among the three learned professions is not considerable, 3. That the three learned professions occupy the following relative position in respect to the duration of their lives, the longest being placed first: medical men, clergymen, lawyers. The vital statistics of Boston show that "gentlemen," or those living on their incomes, are still longer lived than either professional men or farmers.—*Med. & Surg. Reporter.*

---

*Ancient Anæsthesia.*—It appears the Chinese employed anæsthesia long ago. In a biographical notice of Hoa-tho, who flourished under the dynasty Wei, between the years 230 and 340 of our era, it is stated that he gave the sick a preparation of cannabis (*Ma-yo*), who in a few moments became as insensible as one plunged in drunkenness or deprived of life; then, according to the case, he made incisions, amputations, and the like. After a certain number of days, the patient found himself re-established, without having experienced during the operation the slightest pain. It appears from the biography of Han that this cannabis was prepared by boiling and distillation.—*Surg. & Med. Reporter.*

---

*Treatment of Cancrum Oris.*—Of all the local remedies or applications I have resorted to in such cases, I have never found any application so useful or so effectual as hydrochloric acid. Neither nitric acid, nitrate of silver, nor chlorate of potash, nor any other remedy that I have ever tried, except hydrchloric acid, did I ever find of the least use to check cancrum oris. I have almost never found hydrochloric acid to fail to check the progress of this dreadful disease at once, and bring on a most rapid and healthy action in the part, Nor does it cause so much pain or suffering to the little patient as one would suppose, seeing that the gamgrenous spot is almost entirely without feeling at this time. This acid is easily applied to the ulcer by means of a feather or small camel hair brush. I have cured many cases of cancrum oris by this means.—*Dr. McGreevy in British Med. Journal.*

